# Theory of change: Drama and arts-based community engagement for malaria research and elimination in Cambodia

**DOI:** 10.12688/wellcomeopenres.16574.2

**Published:** 2021-05-12

**Authors:** Mom Ean, Nou Sanann, James J. Callery, Christopher Pell, Thomas J. Peto, Rupam Tripura, Phaik Yeong Cheah

**Affiliations:** 1 Mahidol Oxford Tropical Medicine Research Unit, Faculty of Tropical Medicine, Mahidol University, Bangkok, Thailand, 10400, Thailand; 2 University Research Company, Phnom Penh, Cambodia; 3 3Amsterdam Institute for Global Health and Development, Amsterdam, The Netherlands; 4 Centre for Tropical Medicine and Global Health, Nuffield Department of Clinical Medicine, University of Oxford, Oxford, UK; 5 The Ethox Centre, University of Oxford, Oxford, UK

**Keywords:** Malaria, engagement, ethics, drama, arts, Cambodia, Theory of Change

## Abstract

**Background**: Across the Greater Mekong Sub-region, malaria persists in isolated communities along international borders. Arts and drama have been used to reach to communities in Cambodia to engage them in malaria research, prevention and control. The “Village Drama Against Malaria” (VDAM) project was conducted in north eastern and western Cambodia: Stung Treng; Battambang and Pailin provinces during 2016 to 2019.  In total, VDAM reached 55 rural villages, 2,378 student participants and 43,502 audience members.

**Methods**: This article presents the results of two stakeholder-led evaluation workshops in which participants collaboratively developed theories of change to better understand the potential and actual impact of arts and drama-based activities on malaria in these communities. The workshops had a particular focus on identifying areas for monitoring and evaluation so that impact can be measured. Workshop participants included village malaria workers, community leaders, professional and student drama performers, and representatives from the local health authorities and the national malaria control programme.

**Results**: Five broad areas were identified as relevant for monitoring and evaluation: logistical and practical challenges; embeddedness and reach of engagement; health knowledge and confidence of young people; effectiveness of communications; impact on malaria. These areas align well with the monitoring and evaluation conducted to date and point to additional opportunities for data collection.

**Conclusions**: The findings from these workshops will inform future engagement strategies, for example, we may engage a smaller number of young people but over a longer period and more in-depth.

## Introduction

### The public health threat of malaria in Cambodia

Malaria incidence and related mortality declined in Cambodia during the 2010s ^1^. With greater dedicated funding, there were notable improvements in prevention and control, specifically improved case detection and increased availability of insecticide treated bed nets (ITNs) and effective anti-malarial drugs ^2, 3^. Economic development, including changes in land-use patterns, have probably also contributed. During 2017–18 there was a transient increase in transmission. Since then, cases have declined sharply, especially falciparum malaria. Many previously malarial provinces are now free of malaria or approaching the interruption of local transmission and zero deaths from malaria have been reported in the past three years ^4^. However, continued dedication to eliminating malaria and new strategies to reach remote populations are necessary to complete the task ^5^. Multidrug resistant *Plasmodium falciparum* strains have also emerged and spread ^6– 8^, and with no alternative drugs to replace artemisinin-based therapies (ACTs) as first-line treatment, this could have a severe public health impact, particularly in areas with high malaria-related morbidity and mortality ^9, 10^.

In Cambodia, and the wider Greater Mekong Sub-region (GMS), malaria parasite reservoirs tend to cluster along international borders and around forests ^11– 13^. In these areas, malaria remains endemic in high-risk populations, including mobile migrant workers and forest goers ^14– 16^, and asymptomatic carriage plays a role in maintaining transmission ^12^. The communities where these groups reside are often geographically isolated and home to ethnic minorities ^17, 18^. Novel strategies are therefore needed to reverse these recent set-backs: for example, mass antimalarial drug administration has been piloted to address asymptomatic transmission ^19, 20^ and innovative programmes such as those employing art and theatre have sought to engage hard-to-reach communities in malaria prevention and control ^21– 23^.

### Evaluating drama and arts-based community engagement around malaria

Since 2016, together with Cambodia’s national centre for malaria prevention and control (CNM), the Mahidol Oxford Tropical Medicine Unit (MORU) has organized a series of malaria-related community drama and arts events, “Village Drama Against Malaria” (VDAM) in villages in the provinces of Battambang (2016), Pailin (2017) and Stung Treng (2018 and 2019). No events were organized in 2020 due to coronavirus disease 2019 (COVID-19). In parallel, the project team has collected information to monitor and evaluate the community engagement activities, particularly in terms of local participation and stakeholder experiences. The activities have been well received by the communities involved: attendances have been high, with people attending from surrounding villages. Interview respondents, including audience members, community leaders and performers, have been very positive about the events ^21, 22^. Involving students and integrating local dance and costume were elements of the performances that people particularly valued ^23^.

Involving local stakeholders in the process of defining the criteria for project monitoring and evaluation is an important step towards their full engagement ^24^. “Theory of change” (TOC) approaches emphasize techniques that are collaborative, participatory, and practical or applied. As such, TOC was well aligned with our goal of co-creating a monitoring and evaluation framework. Our past evaluations of the VDAM project have been led by researchers and elicited stakeholders’ perspectives on particular activities and their challenges ^21^ but little emphasis has been placed on how they understand the potential benefits and how they see these benefits being achieved in terms of the longer term goal of malaria elimination in Cambodia. To garner their input, with a view reflecting on the VDAM activities from 2016 to 2019 and to plan community engagement activities in the future, we conducted workshops with stakeholders involved in the VDAM programmes. Workshop participants collaboratively developed theory of change frameworks to better understand the (potential and actual) impact of arts and drama-based activities from 2016 to 2019 on malaria in the communities as well as the wider context in which the activities occur. This article presents the results of these workshops, and discusses their relevance for monitoring and evaluation.

## Methods

### Theory of change

A theory of change aims to describe explicitly how a project or initiative can achieve the intended outcomes, considering its context ^25^. It provides a framework to articulate the complex pathways that lead from activities to specific outcomes and the ultimate impact. The assumptions that must be met for the activities to produce the intended outcomes and impact are also outlined. The theory of change is also used to identify indicators that can be used to assess the progress of any programme toward achieving the intended outcomes and aims ^26^.

### Settings

The VDAM project was conducted in the north eastern and western Cambodia: Stung Treng; Battambang and Pailin provinces from 2016 to 2019. These areas are close to the Lao PDR (Stung Treng) and Thailand (Battambang and Pailin) borders, and are predominantly rural. They report differing trends in malaria transmission: Stung Treng has one the highest malaria incidence in Cambodia, whereas Battambang and Pailin form part of the Thai–Cambodia border region that was targeted as a priority areas for the containment of artemisinin-resistance and has seen recent declines in malaria incidence ^27^. The number of malaria cases in Stung Treng was among the top 10 of 43 operational districts in 2020, as calculated using the Annual Blood Examination Rate ( https://mis.cnm.gov.kh/Dashboard/V2).

In all these locations, livelihood activities generally involve subsistence agricultural and forest going. These villages have only basic infrastructure and very little modern entertainment. Strung Treng has a more ethnically diverse population, with notable Kaviet and Lao populations.

At the time of the VDAM activities, clinical studies were underway as part of a long-standing collaboration between MORU and the National Malaria Control Programme of Cambodia (CNM) ^19, 28, 29^. However, the Strung Treng field site was more recently established compared to the other field sites.

### The “Village Drama Against Malaria” approach

The programme was undertaken in Battambang and Pailin Provinces ^21, 22, 30^ in 2016 and 2017, and then in Stung Treng Province in 2018 and 2019 ^23^. The programme ran alongside other engagement work conducted by local health authorities, NGOs and malaria researchers. Throughout the planning and implementation, the project entailed collaboration between key local health staff, community leaders, performers and MORU engagement/research staff. Although malaria (prevention and early treatment) was the main topic of the community engagement, additional health domains were identified as priorities in consultation with local stakeholders. These varied from year to year, and included infant vaccination and antenatal care although these were not MORU research themes. In light of the relatively low literacy rates (compared to urban areas), print media, such as leaflets and posters, was deemed inappropriate and drama was selected as the central activity. In addition, some communities especially in Stung Treng province do not speak Khmer language, but only ethnic languages. In each year, the total duration of the programme was two to three months, and consisted of several stages:

During an orientation and consultation meeting, the activities, target villages and topics for the engagement activities were discussed with local stakeholders (including commune and village leaders, primary school principals, the police chief, the heads of health centres and village malaria workers). The VDAM project team and drama performers also participated. Attendees were given the opportunities to shape the community engagement plans. The project team could obtain feedback and adapt the plan and key messages. Several informal meetings between the project team, drama performers and the community followed to finalise the engagement activities.

1. Over three days in each village, the drama team conducted art and theatre workshops with local children. For most children, this was the first time they have had the opportunity to participate in a theatre workshop. Project staff first met with commune and village leaders, the director and teachers of the local school and village malaria workers to discuss the activities and invite students to participate. With parental consent and help from school teachers, local students joined drama and dance workshops, and rehearsals to prepare for a village performance. The drama team taught the performers the dialogue and steps and instructed them on the malaria messages to be conveyed. Younger children created drawings related to malaria. In 2019, whilst in the villages, the project team also talked with villagers about local costume, musical instruments, art and dance, which were incorporated into the performances. On the third evening, local children performed alongside the professional drama team in the village square. There were also games, singing competitions, malaria quizzes, a fashion show and a comedy sketch about malaria. All community members were invited to attend the event. In total, Village Drama Against Malaria reached rural 55 villages, 2378 student participants and 43502 audience members. See Table 1 for a summary of participants and audience numbers from 2016 to 2019.

**Table 1.** Number of villages, participants and audience members for the Village Drama Against Malaria project from 2016 to 2019.

Year	Province in Cambodia	Number of villages	Number of *participants	Number of audience members
2016	Battambang	20	300	8620
2017	Pailin	15	600	12,410
2018	Stung Treng	10	955	13,610
2019	Stung Treng	10	523	8842
Total		55	2378	43,502

*Young people involved in the performances (e.g dancing, comedy malaria-themed sketches, fashion show) and competitions

2. At a closing event each year, there were speeches, competitions, performances from professional singers, local traditional dances, and an amateur singing competition. Provincial, commune, district and village leaders along with local institutions and NGOs were invited. Senior staff at the National Center for Parasitology Entomology and Malaria Control (CNM) attended. In Stung Treng, groups from villages previously visited were invited as special guests to perform their local dance and music.

### Ethics and consent

Our workshops did not fall under the definition of human subject research and therefore we did not seek ethics approval. Consent was implied by accepting our invitation to the workshops and participation in the workshops. We also sought specific verbal consent to audio record the sessions. 

### Workshop participants

Diverse stakeholders were invited to participate in two day-long workshops: one in Battambang on 30 ^th^ August 2019 and one in Stung Treng on 2 ^nd^ September 2019. These workshops occurred after the last VDAM events in 2019. Participants were drawn from the provincial malaria control programme, the villages that participated in the drama activities (village leaders, village malaria workers and student performers) and the drama groups that led the performances. They were invited to participate by the project staff at the two MORU research sites by sending an official email or invitation letter. A total of 19 participants accepted the invitation and participated in each workshop.

### Workshop format

During the first session of the workshop, a series of presentations were given by members of the project team and the lead facilitator (CP) who was not directly involved in the day-to-day running of the VDAM project. The presentations included an overview of the current malaria situation in the provinces, results of recent malaria research studies conducted by MORU/CNM and a summary of the VDAM project from 2016 to 2019. The latter, which included photos and videos of the VDAM events served as a reminder to the participants regarding the details of the events, which had taken place over the last four years. A third presentation introduced the idea of a theory of change and explained the different elements involved and the aim of involving them in this process. Emphasis was placed on the relevance of their input as participants in terms of identifying relevant activities, successes, failures, challenges, assumptions and defining the aims of the project as they saw them. Structured questions were posed to guide this discussion. 

All the presentations were given in Khmer or there was direct translation from English to Khmer. Several hours were allocated to the presentations to ensure that there was time for questions, particularly regarding the elements of the theory of change framework.

### Data collection

In groups of four to six, participants worked together, with the assistance of a facilitator, who had not been directly involved in the day-to-day running of the VDAM project and interpreters. A member of each group was nominated as scribe and rapporteur. They drew out the impact pathway diagram based on the discussion and comments from the group.

Based on the theory of change, the groups were asked to draw out an impact pathway diagram using flip charts, post-its notes. This entailed identifying and describing: the different component activities of the project; potential outcomes and impacts of the activities; and the overall aim of the project. With the help of group participants, the scribe drew the relationships between these activities. The group also identified and described the limits of these relationships, assumptions that must be fulfilled to achieve them, and the role of wider context. With the assistance of the facilitator and interpreters, each group created a visual representation of the theory of change – an impact pathway. The diagrams were mostly written in Khmer script and interpreters translated the different elements of the diagrams directly to English to facilitate discussion and the recording process.

At the final session of the day-long workshops, the rapporteur presented the group’s theory of change diagram to the other group members, who – along with the facilitator – were given the opportunity to ask questions about and improve on the elements of the theory of change.

Throughout the workshops, the interpreters and facilitators took notes, and the main sessions were digitally recorded with participant consent. All consent and data security measures adhered to the MORU standard operating procedures. The interpreters subsequently checked the recordings against their notes to ensure that any detail or nuance from the discussion had been overlooked. All recordings were destroyed after the notes were taken. The notes were used to supplement the theory of change diagrams for thematic analysis to identify the main areas relevant to monitoring and evaluation for the programme.

## Results

### Theory of change

**Figure 1.**
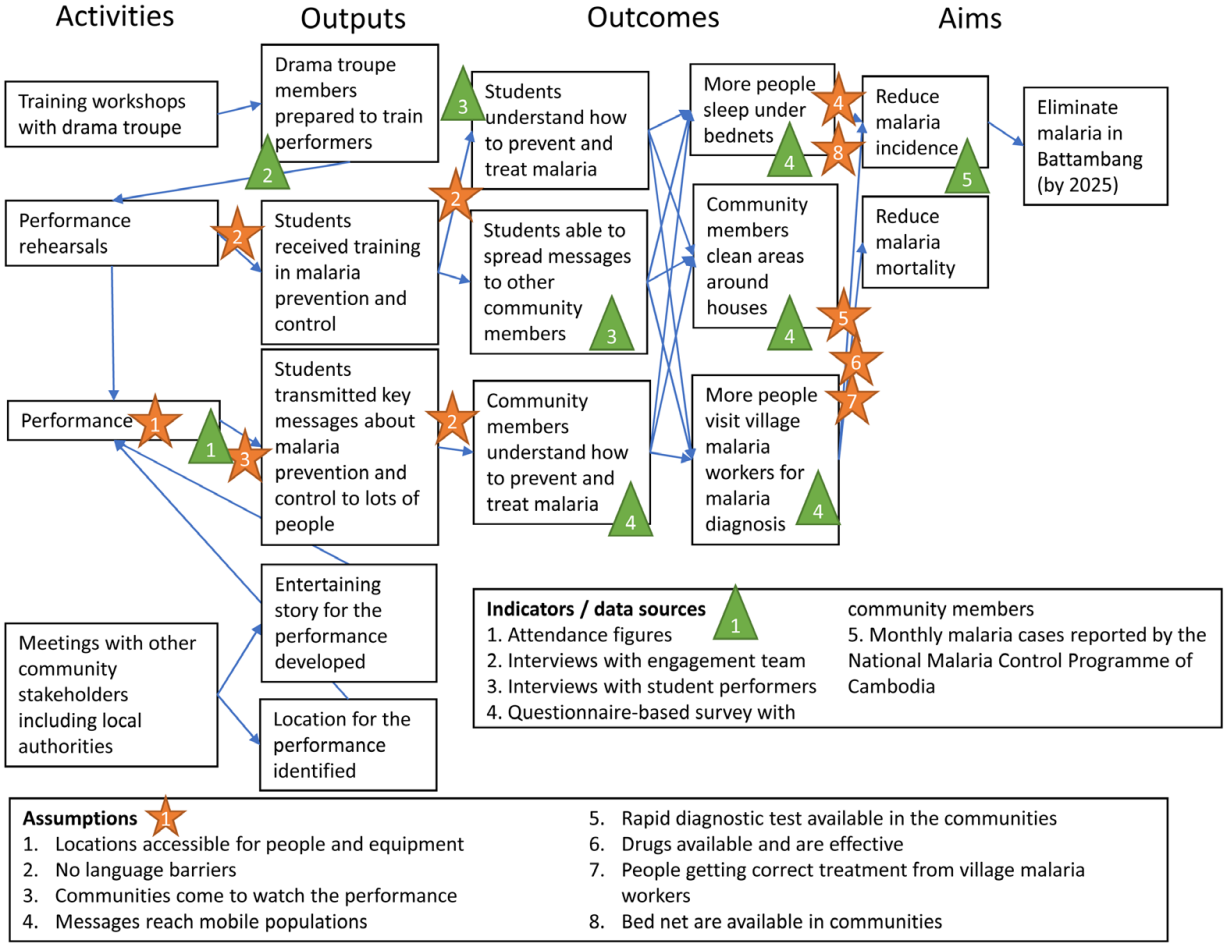
Theory of Change framework, Battambang workshop.

**Figure 2.**
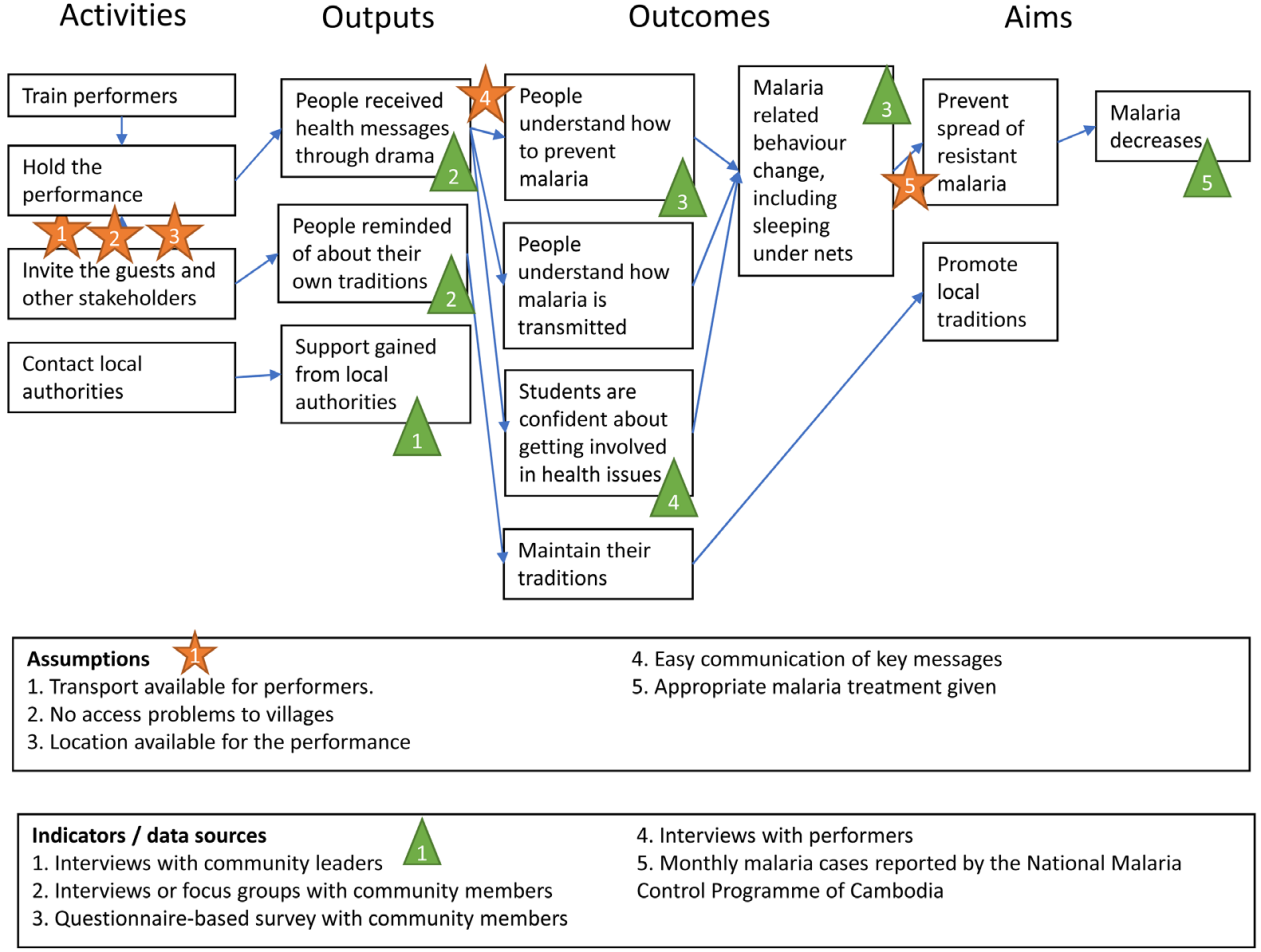
Theory of Change framework, Stung Treng workshop.

Figure 1 and Figure 2 provide a consolidated theory of change from each of the workshops. The TOCs linked the specific activities of the VDAM programme to its ultimate aim, which is to decrease the incidence of malaria in the case of Stung Treng or eliminate malaria in the case of Battambang. The activities or input required included training of student performers, having rehearsals, inviting guests to the performances and dialogues with community stakeholders and local authorities.

Because of the interaction between the groups during each workshop, there were similarities between each group’s diagram and the combined theories of change in the figures are additive versions across the groups of each workshop. There was little direct conflict between the different groups’ diagrams and differences were mainly in the phrasing of particular items. We did not attempt to combine the TOCs for each workshop into one, as there were some differences between them due to additional location specific aims of the VDAM project (e.g. promoting ethnic arts in Stung Treng) and different malaria incidences between provinces.

### Considerations for monitoring and evaluation

Five broad areas were identified from the above TOC frameworks as well as discussions with the workshop participants, as particularly relevant for monitoring and evaluation of the engagement activities, each discussed in turn below.

*1. Logistical and practical challenges of the VDAM project*

During both workshops, participants highlighted the practical and logistical challenges of successfully undertaking the activities in the geographically isolated locations. The project team moved from village to village and only had three days in each village. Workshop participants who were involved directly with organizing the programmes emphasized how preparation was needed to ensure that these challenges could be overcome. The activities required difficult journeys with heavy equipment (e.g. for stage and sound), particularly for the performances. There were also travel issues for audience members such as “crossing river” and “carrying many things” because of the dispersed nature of some settlements, poor or lack of roads and lack of public transport. Because of budget and time constraints, the timetable for activities was demanding and some of the drama team highlighted how this impacted the performances. Garnering feedback from the engagement team and from the performers throughout the running of the programme was seen as one way of gaining an indication of these challenges and mitigate them early on.

****

*2. Level of embeddedness and reach of engagement*

Participants in both workshops recognized the significance of engaging with local leaders and the village authorities prior to and during the drama and arts programme. These meetings were essential to gain the necessary permissions to hold meetings and events in the communities but also brought a crucial added value to the programmes. Drama performers noted how local leaders had helped them to create an engaging and locally appropriate script for the performance, that complements existing engagement efforts by local health leaders and NGOs. In addition, this is important to ensure that our activities add value to existing efforts as well as not inadvertently undermine them. Engaging prominent local figures in this way also meant that they were more likely to attend the performances, and this lent legitimacy to the village events. Similarly, the close involvement of the provincial health authority and national malaria control programme ensured the presence of senior figures from these organizations at the yearly closing events. This combined with the attendance of district-level officials brought media attention to the events. Indicators for the extent of this engagement included the number of participants at events, media coverage and the presence of community leaders. Interviews with community leaders was also a way to assess the role of this broader engagement.

*3. Young people’s health knowledge and confidence*

The rehearsals were a key element of the programmes and workshop participants highlighted challenges in terms of preparing the students to perform. These challenges were mainly related to the limited time. During the rehearsals, performers were instructed in malaria prevention and early treatment as part of the messages to communicate during the performance, and communicating the messages effectively was crucial. The preparation and performance had a broader impact, beyond absorbing and regurgitating health messages. At both workshops, participants described how – through the rehearsals and performances – the young people involved gained greater confidence to engage in health issues and spread the messages. This included talking about what they had learnt beyond the drama performances. Assessing the impact on student performers’ knowledge of malaria prevention can be achieved through, for example, a quiz, questionnaire or interview, however any effect on their confidence as well as other long term impacts is less quantifiable, and challenging to evaluate in the short term.

*4. Effectiveness of communication*

Effective communication, without language barriers, was mentioned as key when putting across the messages to the wider community during the performance. Communication as a potential challenge was a mentioned more in the Battambang workshop despite a greater linguistic diversity in Stung Treng. In addition, participants also noted, “message needs to reach mobile population”, referring to forest workers who travel from place to place to collect wood and fruit or hunt. Gaining insight into whether these outcomes are achieved requires surveying the audience and the wider communities to assess their comprehension of the messages conveyed and their awareness of malaria prevention and management.

*5. Impact on malaria*

Workshop participants saw the programmes as aspiring to community-wide benefits, in terms of malaria prevention, control and ultimately elimination (not just for the young people involved directly in the performances). This related to knowledge about malaria and behaviour change, such as “sleeping under bednets when go to forest” and “clean surrounding areas”.

Groups at both locations focused on quite general aims linked to reducing or eliminating malaria. The Battambang group explicitly mentioned elimination (though this reflects the lower incidence of malaria in this area making this a realistic goal). The groups focused on having an impact on malaria, though in Strung Treng participants also mentioned the aim of support local arts and crafts. This reflects the addition of local costume and dance to the performances in Stung Treng. No connection was mentioned between the drama and arts programme and recruitment in the clinical research that was ongoing at the research sites. Malaria incidence fell markedly during the period of this work, but this also happened in other regions of Cambodia and it is not possible to infer a direct impact attributable to these activities.

A range of assumptions that need to be filled to ensure impact were identified outside the immediate control of the drama and arts programme. The Battambang workshop participants particularly noted a range of structural barriers, such as adequate testing and availability, effective antimalarials, and the importance of the village malaria workers. Here, indicators of success are available among data collected routinely as part of the national malaria control programme (though the strength of the village malaria worker network is also relevant to the quality and coverage of this information).

## Discussion

Through the theory of change workshops, we sought to involve stakeholders in characterizing evaluation criteria for the Village Drama Against Malaria project in Cambodia. This is part of promoting greater engagement, whereby stakeholders not only provide data as part of monitoring and evaluation but rather also influence the nature of the data that are collected and how success is defined. It is hoped that the active involvement of stakeholders will increase ownership and sustain interest in engagement activities.

During the workshops, in groups, stakeholders developed theories of change. Because of interactions between the groups in each workshop, the theories of change were consolidated in the results. There were similarities between the consolidated theories of change from the two workshops, though more complex impact pathways were generated in Battambang ( Figure 1). Five broad areas were identified as relevant for monitoring and evaluation: logistical and practical challenges; embeddedness and reach of engagement; health knowledge and confidence of young people; effectiveness of communication; impact on malaria. These areas align well with the monitoring and evaluation that MORU-CNM has conducted to date but also point to additional opportunities for assessment that are important to stakeholders. They also highlight the limits of the impact that the drama and arts programme can have on malaria indicators.

Formal interviews have not been undertaken with the VDAM team; rather feedback on the challenges has be gathered through team meetings and informal discussions. During these meetings and discussions, the challenges mentioned in the workshop were also described. This was particularly the case when the rehearsals and performances coincided with the rainy season ^21^. After the performances each year, formal interviews and focus groups were conducted with community members, including leaders. Informal feedback was also garnered during the programmes. Collecting information alongside the programme enabled the team to respond to the challenges that the team faced and to adapt much as possible within the budgetary limits. One important example was the suggestion of involving more student performers alongside the professional drama team. During future programmes, this process could be more systematic, with the feedback and responses recorded alongside the programme.

As elsewhere, workshop participants highlighted the importance of step-wise engagement, first involving community leaders and later the wider community ^31, 32^. During previous evaluations, interviews with community leaders, village malaria workers and health care staff had focused on their experiences of the performances and whether they saw the drama and arts as an effective strategy to communicate the health-related messages. In these interviews, there was a general appreciation for this approach. Relatively little emphasis was placed on the process of preparing for the drama and the engagement with community leaders beforehand. The results from the workshops indicate that this should be added to future evaluations. The positive attitudes towards the drama and arts was reflected in the high attendance figures – information that has been collected as part of the previous monitoring and evaluation ^21, 30^.

After the drama and arts programmes, in 2016 and 2018, the teams conducted interviews with performers to garner information about their experiences of performing and what they had learnt during the process of rehearsal and performance. These interviews highlighted the benefits that the workshop participants posited and the performed described being confident to talk about what they had learnt after the performances, particularly with their performance ^21– 23^.

One of the goals of the interviews conducted after the drama and arts was to understand its community-level impact, particularly in terms of how it impacted people’s awareness of malaria prevention and control ^23^. It is likely that VDAM consolidated knowledge of malaria in the target communities. Interviews were conducted before and after the programme but community members demonstrated a high initial awareness of malaria, which made it difficult to discern the impact of the malaria-related health messages. Community members were however able to recall many of the messages that formed part of performances. Respondents also reflected on the limitations of the impacts of these messages: for example, efforts to reduce contact with mosquitoes was not limited by knowledge but rather by livelihood activities in the forests ^17^. This highlights the importance of formative research to assess initial awareness of the health topics in the wider community and adjust the programme appropriately.

Given the range of additional determinants, as identified by workshop participants, assessing the programme’s impact on local malaria incidence or prevalence is difficult. Health system factors, such as the availability and accessibility of testing and appropriate treatment play a crucial role in malaria incidence and prevalence, as do the availability of bednets. For example, in Cambodia, the withdrawal of Global Fund support for the village malaria workers in 2016–2017 has been linked to the subsequent large increase in reported malaria cases ^33^. By contrast, in most parts of Battambang, incidence decreased to almost zero in the years after the drama and arts programme. This coincided also with mass antimalarial drug administration programmes in these areas. One must therefore be careful when evaluating programmes such as this in terms of malaria indicators, whether at a local or larger scale.

Notably, when asked about the aims of the drama and arts programme, the participants made no mention of recruitment to the clinical studies underway at the local study sites. However, the malaria researchers felt that VDAM activities had a positive impact in sensitizing communities to research projects and thereby making it easier to recruit participants into studies especially large clinical trials. This could be because the VDAM project was conducted in both villages which were not involved in malaria clinical studies and those which were not. In some years, the VDAM project also included health topics unrelated to malaria research. While this is positive in terms of public health, it also likely highlights diverging priorities between the researchers and other stakeholders. In addition to malaria prevention and control, the VDAM project aimed to support the clinical studies ongoing at the time of the project. Our paper also indicates a need to place greater emphasis on the role of the clinical studies of addressing malaria and reaching the goals of malarial elimination in future programmes of engagement in the research sites.

These workshops as well as previous evaluations highlight several limitations of the VDAM as an engagement project. The project was successful in reaching large numbers ( Table 1) of audience members with low literacy, and helped spread awareness on malaria control and prevention. It also informed communities of ongoing malaria research projects. However, the level of interaction with students and local stakeholders in each village was low. Many VDAM challenges such as lack of time for rehearsals were due to time constraints and the team having to travel from village to village in a short period of time. Strategies that engage young people in more depth and enabling them to contribute to research, such as “young persons’ advisory groups (YPAGs) may be employed in the future. Elsewhere in Cambodia, YPAGs have been shown to be an effective way to engage young people ^34^.

The study had several strengths. The workshops were facilitated by the project team, but input and the elements of the TOC framework were obtained from the participants. It reports the process and results of a theory of change approach developed by relevant and diverse stakeholders that identifies areas of evaluation for our VDAM programme and future engagement programmes. Most workshop participants have lived and worked in the relevant provinces in Cambodia and are knowledgeable about the malaria situation, the context, VDAM and other engagement activities in the area.

The findings are limited by the duration of the TOC workshops, for which only one day was possible in each location because of busy schedules of participants and the need to travel to a central location. In addition, due to translations (from Khmer to English), some of the nuances could have been missed. In each workshop, participants were drawn from senior roles in district and provincial government as well as the general public in the intervention villages. The workshop facilitators have years of experience in facilitating workshops and made every effort to be inclusive. However, it was possible that the views of some participants might not have been fully represented.

## Conclusion

Using a theory of change approach, at two workshops, stakeholders involved in the drama and arts programmes in western and northern Cambodia developed impact pathways to link their activities with the ultimate goals. The aim of the workshops was to involve stakeholders in characterizing evaluation criteria for future programmes. Five broad areas were identified as relevant for monitoring and evaluation: logistical and practical challenges; embeddedness and reach of engagement; health knowledge and confidence of young people; effective communication; impact on malaria. These areas align well with the monitoring and evaluation that MORU-CNM has conducted to date. Workshop participants also identified a range of additional determinants of local malaria indicators and this highlights the difficulties in assessing the programme’s impact in this regard. COVID-19 has prevented the team from holding VDAM events in 2020, however, the findings from these workshops will inform future engagement strategies, for example, we may engage a smaller number of young people but more in-depth.

## Data availability

### Underlying data

Workshop notes are available upon request to the MORU Data Access Committee by completing a Data application form.
