# Supplementary material for: Theory of change: Drama and arts-based community engagement for malaria research and elimination in Cambodia
Source: Wellcome Open Res. 2021 May 12;6:46. Originally published 2021 Mar 2. [Version 2] doi: 10.12688/wellcomeopenres.16574.2 (PMC8127021; doi:10.12688/wellcomeopenres.16574.2)
Supplement: Peer review history

# Peer review: Reviewer response for version 2

**DOI:** 10.21956/wellcomeopenres.18618.r43850

**Published:** 2021-05-12

**Authors:** Lucinda Manda-Taylor

**Affiliations:** 1 School of Public Health and Family Medicine, College of Medicine, University of Malawi, Blantyre, Malawi

Thank you to the authors for responding to the issues I raised. I now approve the publication of this version of the manuscript.

Is the work clearly and accurately presented and does it cite the current literature?

Partly

If applicable, is the statistical analysis and its interpretation appropriate?

Not applicable

Are all the source data underlying the results available to ensure full reproducibility?

Yes

Is the study design appropriate and is the work technically sound?

Yes

Are the conclusions drawn adequately supported by the results?

Yes

Are sufficient details of methods and analysis provided to allow replication by others?

Yes

Reviewer Expertise:

Bioethics and Social Science

I confirm that I have read this submission and believe that I have an appropriate level of expertise to confirm that it is of an acceptable scientific standard.

# Peer review: Reviewer response for version 1

**DOI:** 10.21956/wellcomeopenres.18266.r43204

**Published:** 2021-03-29

**Authors:** Lucinda Manda-Taylor

**Affiliations:** 1 School of Public Health and Family Medicine, College of Medicine, University of Malawi, Blantyre, Malawi

1. In the second paragraph, under the heading, **Evaluating drama and arts-based community engagement around malaria, **the acronym TOC is used. Please make sure that when it is the first time the abbreviation is being presented that it is written out in full. 
2. The authors state that, "These areas report differing trends in malaria transmission. Stung Treng has one of the highest malaria incidences in Cambodia. In contrast, Battambang and Pailin form part of the Thai–Cambodia border region that was targeted as priority areas for the containment of artemisinin-resistance and has seen recent declines in malaria incidence". It may be useful to include data on the incidence and/or prevalence of malaria in these areas/regions. This evidence should be presented against malaria's incidence or prevalence in the other regions in the country. 
3. The authors note that this type of study did not require formal ethics approval under ethics and consent. A statement on the local ethical and regulatory guidelines that exempt them would be useful to determine that, indeed, local guidelines of human subjects protection were sought. A statement on the above would help validate their assertion that this does not fall under the definition of HSR. 
4. There is a letter missing from this sentence to make it grammatically correct. "Garnering feedback from the engagement team and from the performers throughout the running of the programme **was **seen as one way of gaining an indication of these challenges and mitigate them early on".
5. The authors state that consent to participate in the workshops was implied. Yet, further down they write the main sessions were digitally recorded with participant consent. Was the consent expressed, or implied? 
6. Of value, would have been the addition of specific quotes from participants to support the findings. Can the authors explain why the voices of the workshop participants are missing since consent to record the discussions was either expressed or implied?
7. Were there any structured questions that guided the discussion in the stakeholder workshops that enabled the generation of themes? 
8. Re: Discussion, paragraph 5: Given that the VDAM programme rang alongside other engagement work conducted by local health authorities, NGOs and malaria researchers, do the author's think that this may have contributed to the already existing knowledge on malaria in the community and that arts and drama simply complemented the existing community engagement work?
9. Theme 4 seems to focus much on communication as a key requisite for messaging that could help improve knowledge on malaria and improve community health outcomes. I would suggest the authors revisit this theme and consider revising it.
10. How would the author's appraise their approach to community engagement? Do the authors feel that the approach was a bottom-up, participatory approach, or was it a top-down approach or a mixture of the two? How does the approach they used influence the results?
11. The manuscript should be proof-read to check and improve on the grammatical errors.

Is the work clearly and accurately presented and does it cite the current literature?

Partly

If applicable, is the statistical analysis and its interpretation appropriate?

Not applicable

Are all the source data underlying the results available to ensure full reproducibility?

Yes

Is the study design appropriate and is the work technically sound?

Yes

Are the conclusions drawn adequately supported by the results?

Yes

Are sufficient details of methods and analysis provided to allow replication by others?

Yes

Reviewer Expertise:

Bioethics and Social Science

I confirm that I have read this submission and believe that I have an appropriate level of expertise to confirm that it is of an acceptable scientific standard, however I have significant reservations, as outlined above.

# Response

**Published:** 2021-04-27

**Authors:** Phaik Yeong Cheah

**Affiliations:** Mahidol Oxford Tropical Medicine Research Unit, Thailand

*Thank you for valuable comments. Below is a point by point response.*

 In the second paragraph, under the heading, Evaluating drama and arts-based community engagement around malaria, the acronym TOC is used. Please make sure that when it is the first time the abbreviation is being presented that it is written out in full. 

*Thank you. We have now spelled out TOC.*

 The authors state that, "These areas report differing trends in malaria transmission. Stung Treng has one of the highest malaria incidences in Cambodia. In contrast, Battambang and Pailin form part of the Thai–Cambodia border region that was targeted as priority areas for the containment of artemisinin-resistance and has seen recent declines in malaria incidence". It may be useful to include data on the incidence and/or prevalence of malaria in these areas/regions. This evidence should be presented against malaria's incidence or prevalence in the other regions in the country. 

*Thank you for the suggestion. We have added a sentence in “Settings”.*

*“The number of malaria cases in Stung Treng was among the top 10 of 43 operational districts in 2020, as calculated using the Annual Blood Examination Rate ( https://mis.cnm.gov.kh/Dashboard/V2)”*

 The authors note that this type of study did not require formal ethics approval under ethics and consent. A statement on the local ethical and regulatory guidelines that exempt them would be useful to determine that, indeed, local guidelines of human subjects protection were sought. A statement on the above would help validate their assertion that this does not fall under the definition of HSR. 

*We did not seek ethics approval as these were workshops, not human subject research. We have many years of experience conducting research as well as workshops in Cambodia.*

 There is a letter missing from this sentence to make it grammatically correct. "Garnering feedback from the engagement team and from the performers throughout the running of the programme **was **seen as one way of gaining an indication of these challenges and mitigate them early on".Thank you. Correction made.

*The authors state that consent to participate in the workshops was implied. Yet, further down they write the main sessions were digitally recorded with participant consent. Was the consent expressed, or implied? Consent was expressed verbally. We edited the ethics paragraph and added a sentence.*

*“We also sought specific verbal consent to audio record the sessions.”*

 Of value, would have been the addition of specific quotes from participants to support the findings. Can the authors explain why the voices of the workshop participants are missing since consent to record the discussions was either expressed or implied?

*We have now added some quotes from post-it notes in the “Considerations for monitoring and evaluation” section. We obtained consent to record the session but not to quote participants directly.*

 Were there any structured questions that guided the discussion in the stakeholder workshops that enabled the generation of themes? 

*Yes there were. We have now added a sentence in “Workshop format”.*

 Re: Discussion, paragraph 5: Given that the VDAM programme rang alongside other engagement work conducted by local health authorities, NGOs and malaria researchers, do the author's think that this may have contributed to the already existing knowledge on malaria in the community and that arts and drama simply complemented the existing community engagement work?

*Yes, we believe so but it was difficult to proof. We have added a sentence in Discussion paragraph 5.*

*“We believe that VDAM contributed to existing knowledge of malaria among community members. Interviews were conducted before and after the programme but community members demonstrated a high initial awareness of malaria, which made it difficult to discern the impact of the malaria-related health messages.”*

 Theme 4 seems to focus much on communication as a key requisite for messaging that could help improve knowledge on malaria and improve community health outcomes. I would suggest the authors revisit this theme and consider revising it.Thank you for the suggestion. We have now rewritten theme 4 and given it a new heading.

*Effectiveness of Communication*

*Effective communication, without language barriers, was mentioned as key when putting across the messages to the wider community during the performance. Communication as a potential challenge was a mentioned more in the Battambang workshop despite a greater linguistic diversity in Stung Treng. In addition, participants also mentioned “message needs to reach mobile population”, referring to forest workers who travel from place to place to collect wood and fruit or hunt. Gaining insight into whether these outcomes are achieved requires surveying the audience and the wider communities to assess their comprehension of the messages conveyed and their awareness of malaria prevention and management.*

 How would the author's appraise their approach to community engagement? Do the authors feel that the approach was a bottom-up, participatory approach, or was it a top-down approach or a mixture of the two? How does the approach they used influence the results?

*We consider this approach a mixture of bottom-up and top-down. we facilitated the theory of change discussion and asked the questions. However, it was the participants who filled in the elements of the TOC framework.*

 The manuscript should be proof-read to check and improve on the grammatical errors.

*We have done that.*

**

*Thank you*

**

*We have also made minor changes throughout the manuscript to improve its clarity and added a few references. The references were added as “comments” as we are unable to add it directly in endnote.*

*Thank you.*

**

*Phaik Yeong Cheah, corresponding author*

# Peer review: Reviewer response for version 1

**DOI:** 10.21956/wellcomeopenres.18266.r42902

**Published:** 2021-03-22

**Authors:** Maxine Anne Whittaker

**Affiliations:** 1 College of Public Health, Medical and Veterinary Sciences, James Cook University, Townsville, Qld, Australia

Thank you for this well-written, and interesting paper reporting on important innovations in both malaria elimination but also in community engagement and participation.

 As a reader I have a couple of follow-up queries:

- do you believe this approach of engagement in the TOC workshop has also increased community engagement/ownership and will support sustainability of interest?
- how have you used the TOC and M and E framework derived there from in your work?

One minor edit:

- page 5 LH column last para - remove on the of "the" "took notes and the main sessions were..."

Is the work clearly and accurately presented and does it cite the current literature?

Yes

If applicable, is the statistical analysis and its interpretation appropriate?

Yes

Are all the source data underlying the results available to ensure full reproducibility?

Yes

Is the study design appropriate and is the work technically sound?

Yes

Are the conclusions drawn adequately supported by the results?

Yes

Are sufficient details of methods and analysis provided to allow replication by others?

Yes

Reviewer Expertise:

global public health, malaria control and elimination, community/civil society engagement; One health, health systems and policy

I confirm that I have read this submission and believe that I have an appropriate level of expertise to confirm that it is of an acceptable scientific standard.

# Response

**Published:** 2021-04-27

**Authors:** Phaik Yeong Cheah

**Affiliations:** Mahidol Oxford Tropical Medicine Research Unit, Thailand

We are grateful for the reviewer’s comments. Below is a point by point response to the reviewer’s comments.

 Thank you for this well-written, and interesting paper reporting on important innovations in both malaria elimination but also in community engagement and participation.

 As a reader I have a couple of follow-up queries:

 1. Do you believe this approach of engagement in the TOC workshop has also increased community engagement/ownership and will support sustainability of interest?

*Yes, we believe so. We have added a sentence in Discussion, para 1.*

*"It is hoped that the active involvement of stakeholders will increase ownership and sustain interest in engagement activities."*

 2. How have you used the TOC and M and E framework derived there from in your work?

*No, we haven’t as our activities in Cambodia were paused due to COVID.*

 One minor edit:

 3. page 5 LH column last para - remove on the of "the" "took notes and the main sessions were..."

*Done*.
